# High frequency chest wall oscillation for asthma and chronic obstructive pulmonary disease exacerbations: a randomized sham-controlled clinical trial

**DOI:** 10.1186/1465-9921-12-120

**Published:** 2011-09-10

**Authors:** Amit K Mahajan, Gregory B Diette, Umur Hatipoğlu, Andrew Bilderback, Alana Ridge, Vanessa Walker Harris, Vijay Dalapathi, Sameer Badlani, Stephanie Lewis, Jeff T Charbeneau, Edward T Naureckas, Jerry A Krishnan

**Affiliations:** 1 Department of Medicine, Section of Pulmonary and Critical Care, University of Chicago, 5841 S. Maryland Ave, Chicago, Illinois, 60637, USA; 2 Department of Medicine, Division of Pulmonary and Critical Care Medicine, Johns Hopkins University, 1830 E. Monument, 5th Floor, Baltimore, Maryland, 21205, USA; 3 Department of Medicine, Mercy Hospital and Medical Center, 2525 S. Michigan Avenue, Chicago, Illinois 60617, USA; 4 Respiratory Institute, Cleveland Clinic, MC A90, 9500 Euclid Avenue, Cleveland, OH 44195, USA; 5 Section of Hospital Medicine, University of Chicago, 5841 S. Maryland Ave, Chicago, Illinois, 60637, USA; 6 Department of Medicine, Section of Pulmonary, Critical Care, Sleep, and Allergy, University of Illinois at Chicago, 840 S. Wood Street, Chicago, Illinois 60612, USA

**Keywords:** asthma, chronic obstructive pulmonary disease, high frequency chest wall oscillation, airway mucus clearance

## Abstract

**Background:**

High frequency chest wall oscillation (HFCWO) is used for airway mucus clearance. The objective of this study was to evaluate the use of HFCWO early in the treatment of adults hospitalized for acute asthma or chronic obstructive pulmonary disease (COPD).

**Methods:**

Randomized, multi-center, double-masked phase II clinical trial of active or sham treatment initiated within 24 hours of hospital admission for acute asthma or COPD at four academic medical centers. Patients received active or sham treatment for 15 minutes three times a day for four treatments. Medical management was standardized across groups. The primary outcomes were patient adherence to therapy after four treatments (minutes used/60 minutes prescribed) and satisfaction. Secondary outcomes included change in Borg dyspnea score (≥ 1 unit indicates a clinically significant change), spontaneously expectorated sputum volume, and forced expired volume in 1 second.

**Results:**

Fifty-two participants were randomized to active (n = 25) or sham (n = 27) treatment. Patient adherence was similarly high in both groups (91% vs. 93%; p = 0.70). Patient satisfaction was also similarly high in both groups. After four treatments, a higher proportion of patients in the active treatment group had a clinically significant improvement in dyspnea (70.8% vs. 42.3%, p = 0.04). There were no significant differences in other secondary outcomes.

**Conclusions:**

HFCWO is well tolerated in adults hospitalized for acute asthma or COPD and significantly improves dyspnea. The high levels of patient satisfaction in both treatment groups justify the need for sham controls when evaluating the use of HFCWO on patient-reported outcomes. Additional studies are needed to more fully evaluate the role of HFCWO in improving in-hospital and post-discharge outcomes in this population.

**Trial Registration:**

ClinicalTrials.gov: NCT00181285

## Background

Acute asthma and chronic obstructive pulmonary disease (COPD) are exceedingly common, which together account for nearly 1 million hospitalizations each year in the United States alone [1-6]. Beta agonists, anti-cholinergics, and corticosteroids delivered in aerosolized forms (via respiratory inhalers or nebulization) are recommended in the treatment of acute asthma and COPD. These medications rely on deposition into distal airspaces to suppress airway inflammation or promote bronchodilation. Unfortunately, excessive mucous production and impaired airway mucociliaryclearance can lead to airway plugging, and thereby reduce the deposition of and response to aerosolized medications. These considerations highlight the need for therapies that clear airways of mucus in the acute management of asthma and COPD [7-11].

High frequency chest wall oscillation (HFCWO) creates high velocity, low amplitude oscillatory airflows when applied through a pneumatic vest worn over the thorax, and is used for airway mucus clearance in patients with cystic fibrosis, bronchiectasis, and neuromuscular disorders [12-15]. Studies in patients with cystic fibrosis suggest that HFCWO applied via a pneumatic vest is as effective as other modes of airway mucus clearance, including hand-held devices (e.g., flutter devices) and conventional chest physiotherapy[16]. HFCWO offers the advantage that it can be performed in acutely ill patients who may be unable to use hand-held devices effectively, such as early in the course of hospitalization. Moreover, HFCWO can be performed without the assistance from trained health care personnel, and may therefore offer a practical advantage compared to chest physiotherapy. Pneumatic vests worn over the chest, however, may not be acceptable to patients with asthma or COPD with worsening respiratory symptoms. To our knowledge, no studies have examined the use of HFCWO in the management of acute asthma or COPD. The objective of this phase II clinical trial (Chest Wall Oscillation for Asthma and COPD ExacerbaTions [COAT] Trial) was therefore to evaluate the use of HFCWO early in the treatment of adults hospitalized for acute asthma or COPD. To minimize the risk of bias, we included active and sham HFCWO treatment groups and standardized medical management in both treatment groups. Preliminary results of this study were previously reported in the form of an abstract [17].

## Methods

### Recruitment

Adults (age 18 years and older) admitted with a physician-diagnosis of acute asthma or COPD at one of four academic medical centers were screened for this study. The treating physician was contacted to confirm the clinical diagnosis (acute asthma, acute COPD, or acute asthma and COPD) and for verbal consent prior to approaching patients for written informed consent. Inclusion criteria included admission to the inpatient medical service and evidence of airflow obstruction on spirometry (forced expired volume in 1 second/forced vital capacity [FEV_1_/FVC] < 70%) at the time of screening. Exclusion criteria were: more than 24 hours since hospital admission, hospital discharge planned within 24 hours, admission to an intensive care unit, other chronic respiratory diseases (e.g. sarcoidosis), chest wall abnormalities (e.g. severe kyphoscoliosis), chest wall or abdominal trauma/surgery in the past 6 weeks, systemic corticosteroid therapy for 7 or more days prior to hospital admission, indication for systemic corticosteroids other than asthma or COPD, patient unable (e.g. due to illness) or unwilling to provide consent, and previous participation. Institutional review boards at participating institutions approved this study (University of Chicago, and Mercy Hospital and Medical Center in Chicago, Illinois, U.S.A.; Johns Hopkins Bayview Medical Center, and Johns Hopkins Hospital, in Baltimore, Maryland, U.S.A.).

### Baseline evaluation and randomization

Participants completed an interviewer-administered questionnaire about demographics, acute care for asthma or COPD in the past year (hospitalizations, emergency room visits, and courses of systemic corticosteroids), and dyspnea using the modified Borg scale. Spirometry (KoKo^®^; Pulmonary Data Services Instrumentation; Louisville, CO) was performed after providing 2 puffs of albuterol via a metered dose inhaler (MDI) and spacer to measure the post-bronchodilator [post-BD] FEV_1_/FVC and post-BD FEV_1 _% predicted. Participants were then randomized to active or sham HFCWO, stratified by site and diagnosis using permuted blocks to ensure balance across treatment groups.

### Treatment conditions

Active HFCWO (The Vest^® ^Airway Clearance System, Hill-Rom, Inc.; pressure dial settings 4-6 units and frequency 10-12 Hz) consists of an inflatable vest and an air-pulse generator, creating oscillatory chest wall compressions and airflow[13,14]. The sham device had a pressure bypass circuit, which provided a vibratory sensation over the chest without causing airflow oscillation and was indistinguishable from the active HFCWO device in appearance and noise production. Treatments were administered by research assistants over 15 minutes and delivered at 8 AM, 12 Noon, and 4 PM each day after 4 puffs of albuterol MDI, 90 mcg/puff. Each participant was prescribed four treatments (total of 60 minutes). Treatments could be interrupted or discontinued altogether at the discretion of the study participant. Research assistants who helped participants put on and activate the pneumatic vest were not involved in the collection of baseline data or outcomes. Also, treating physicians were not permitted to observe study treatments to avoid changes in care due to unmasking.

Based on national asthma [18] and COPD [19] guidelines, medical management was standardized for all participants. Participants received aerosolized albuterol every 4 hours and every 1-2 hours as needed (2.5 mg/mL via nebulization or 90 mcg/puff via MDI, 4 puffs, at the discretion of treating physicians), systemic corticosteroids daily (prednisone 60 mg by mouth or equivalent intravenous dose of methylprednisolone [48 mg], at the discretion of treating physician), inhaled corticosteroids/long-acting bronchodilator (fluticasone/salmeterol 250 mcg/50 mcg via Diskus^®^) one inhalation twice daily, and supplemental oxygen to keep saturations above 93%. Other medications could be prescribed at the discretion of the treating physician.

### Evaluation after four treatments

We assessed patient adherence to prescribed study treatments (minutes used/60 minutes prescribed) and patient satisfaction with study treatment. Satisfaction items were developed for the study and intended to provide descriptive information rather than serve as an efficacy endpoint so formal methodologies typically used to develop and validate patient-reported outcomes (e.g., item generation, item reduction) were not employed. The satisfaction items were: 1) The study vest was convenient to use; 2) The study vest was easy to use; 3) The study vest was comfortable; 4) The study vest helped me feel better; 5) The study vest helped me breathe better; 6) I felt safe using the study vest; 7) I would recommend the study vest to someone with my type of breathing problem; 8) I want my doctor to prescribe the study vest for me. Participants were asked to use a 5-point scale (strongly agree, somewhat agree, neither agree nor disagree, somewhat disagree, strongly disagree) when rating their satisfaction:

The modified Borg scale was used to collect data about dyspnea after four treatments; a ≥ 1 unit reduction defines a clinically meaningful change[20]. Spontaneously expectorated sputum volume (wet volume) after four treatments was measured. Participants were instructed to expectorate as needed into a study container provided at the baseline visit, which was collected after the fourth treatment. Spirometry was used to measure post-BD FEV_1 _% predicted 15-30 minutes after 2 puffs of albuterol MDI.

Decisions regarding hospital discharge were at the discretion of the treating physicians. Discharge medications were standardized to include prednisone 50 mg daily to complete a 10-day course of systemic corticosteroids, inhaled fluticasone/salmeterol 250 mcg/50 mcg Diskus 1 inhalation twice daily, and inhaled albuterol MDI with spacer 2 puffs every four hours as needed. At a follow-up study visit conducted by telephone, patient-reported respiratory-related acute care at 30 days (additional course of systemic corticosteroids, emergency department visit, or hospitalization for "difficulty breathing, cough, or chest tightness") was assessed.

### Statistical Analysis

The co-primary outcomes were patient adherence and satisfaction with HFCWO immediately after four study treatments. Responses to each satisfaction item were collapsed into agree ('yes' [strongly or somewhat agree] or 'no' [else]). Secondary outcomes after four study treatments were the change in dyspnea (follow-up - baseline Borg score), the proportion with a clinically meaningful change in dyspnea, volume of expectorated sputum, and change in post-bronchodilator FEV_1_% predicted (follow-up - baseline). Length of hospital stay after study treatment and respiratory-related acute care within 30 days of discharge were other secondary outcomes. Wilcoxon ranksum tests, or Chi^2 ^tests were performed, as appropriate, for comparisons between groups. A two-tailed p-value less than 0.05 defined statistical significance. This was a Phase II clinical trial primarily designed to assess patient adherence and satisfaction regarding the early use of HFCWO during acute asthma and COPD, so no formal sample size calculations were performed. Results of this study were intended to provide the information needed for sample size calculations for subsequent studies. Version 9.2 of the SAS System (SAS Institute Inc., Cary, NC) was used for all analyses.

## Results

**Figure 1:**
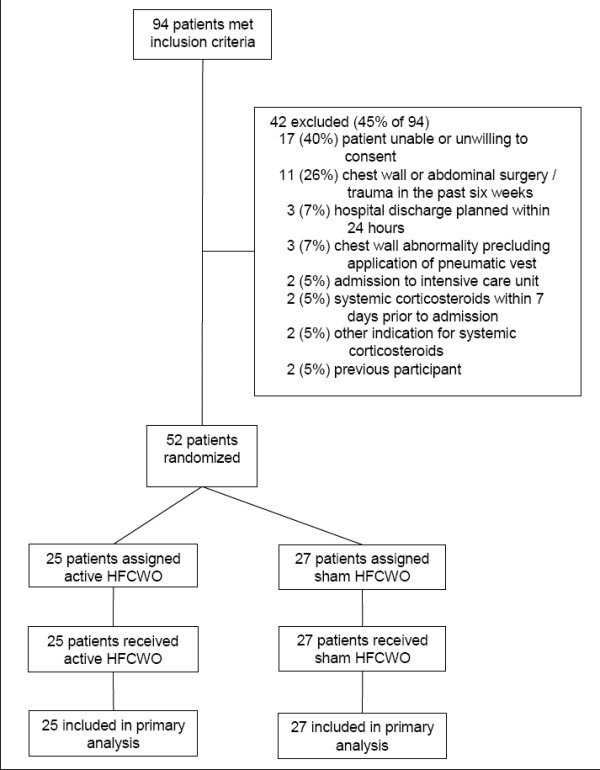
**Flowchart of Study Cohort**. N = 94 adults (age 18 years and older) admitted with a physician-diagnosis of acute asthma or COPD and with FEV_1_/FVC < 70% at the time of screening were assessed for eligibility. Fifty-two (55%) were randomized to active HFCWO (n = 25) or sham HFCWO (n = 27).

Of the 94 patients who met inclusion criteria, 42 (45%) met exclusion criteria (Figure 1). The most common reasons for exclusion were inability to obtain patient consent (e.g., patients were acutely ill and unable to provide written informed consent or patients declined participation, n = 17), chest wall or abdominal surgery or trauma in the past six weeks (n = 11), and hospital discharge planned within 24 hours (n = 3). Fifty-two patients (55% of those who met inclusion criteria) were randomized to receive either active HFCWO (n = 25) or sham HFCWO (n = 27). Nearly two-thirds of study participants had acute asthma. Participants had, on average, one other hospitalization and two previous courses of systemic corticosteroids in the past year. Baseline characteristics were similar in the two treatment groups (Table 1).

**Table 1:** Baseline characteristics of study participants

Characteristic		Active HFCWO (n = 25)	Sham HFCWO (n = 27)	p-value
**Diagnosis, n (%)**	Acute asthma	15 (60)	16 (59)	> 0.99
	Acute COPD	9 (36)	10 (37)	
	Acute asthma and COPD	1 (4)	1 (4)	
**Age, years**		46.5 [38.6, 52.8]	50.4 [43.9, 60.7]	0.28
**BMI, kg/m^2^**		27.0 [23.7, 33.0]	29.7 [23.7, 38.0]	0.43
**Post-BD FEV_1_% predicted**		45 [26, 58] n = 23	40 [33, 55] n = 25	0.75
**Post-BD FEV_1_/FVC, %**		61 [49, 66] n = 23	55 [49, 66] n = 25	0.55
**Hospitalizations past year (excluding current)**		1 [0, 3] n = 21	1 [0, 4] n = 21	0.98
**Emergency room visits past year**		2 [0, 4]	4 [0, 5] n = 25	0.50
**Corticosteroid courses past year**		2 [0, 4] n = 24	2 [0, 5] n = 25	0.58

The median [interquartile range] is reported, unless otherwise stated. The number (n) of participants with data is included in the table, if n is less than the number of participants assigned to each treatment group. Missing data were due to difficulty in performing some tests in acutely ill patients (e.g., post-BD spirometry) or non-response (e.g., problems with patient recall). HFCWO = high frequency chest wall oscillation, BMI = body mass index, Post-BD FEV_1_= post-bronchodilator forced expiratory volume in 1 second, Post-BD FEV_1_/FVC = post-bronchodilator forced expiratory volume in once second/forced vital capacity.

### Primary Outcomes (Table 2)

**Table 2:** Primary outcomes: adherence to treatment and patient satisfaction

	Active HFCWO (n = 25)	Sham HFCWO (n = 27)	p-value
**Adherence, mean (SD)**	91% (21.1%)	93% (18.7%)	0.70
**Satisfaction**			
Convenient	79% n = 24	92% n = 26	0.24
Easy to use	92%	92% n = 26	> 0.99
Comfortable	88%	92% n = 26	0.67
Helped me feel better	80%	85% n = 26	0.73
Helped me breathe	84%	69% n = 26	0.32
Felt safe	100%	96% n = 26	> 0.99
Would recommend to someone	92%	85% n = 26	0.67
Want my doctor to prescribe	76%	81% n = 26	0.74

One participant in the active HFCWO group had missing data for 1 of the patient satisfaction items. One participant in the sham HFCWO group had missing data for all the satisfaction items.

Patient adherence to active and to sham HFCWO was similarly high (91% vs. 93%, p = 0.70) in both groups. Satisfaction with study treatment was also high, even in the sham HFCWO group (active vs. sham HFCWO: comfortable, 88% vs. 92%, p = 0.67; feel better, 80% vs. 85%, p = 0.73).

### Secondary Outcomes (Table 3)

**Table 3:** Secondary outcomes

	Active HFCWO (n = 25)	Sham HFCWO (n = 27)	Comparison between groups p-value
**After four treatments**			
Change in Borg score	-1.5 [-3.5, 0] n = 24	0 [-2, 0] n = 26	0.048
Expectorated sputum, mL	10 [8, 20]	11 [6, 45]	0.44
Change in post-BD FEV_1_% predicted	0 [-2, 8] n = 22	2 [-3, 9] n = 23	0.69
**Length of hospital stay, days**	2 [1, 3]	2 [1, 4]	0.75
**Respiratory- related acute care at 30 days**			
Systemic corticosteroids, n (%)	4 (17) n = 23	2 (8) n = 24	0.42
Acute care visit (hospitalization or ED visit), n (%)	4 (17) n = 23	4 (17) n = 24	> 0.99
Either	5 (22) n = 23	4 (17) n = 24	0.72

The median [interquartile range] is reported, unless otherwise stated. The number (n) of participants with data is included in the table, if n is less than the number of participants assigned to each treatment group. Missing data were due to difficulty in performing some tests in acutely ill patients (e.g., post-BD spirometry) or non-response (e.g., problems with patient recall or inability to respond). There were five participants lost to follow-up (2 in active HFCWO, 3 in sham HFCWO groups).

After four treatments, there was significantly greater improvement in dyspnea in the active HFCWO group (median change in Borg score of -1.5 vs. 0 units, p = 0.048). Nearly twice as many patients reported a clinically meaningful improvement in dyspnea in the active HFCWO group than in the sham HFCWO group (71% vs. 42%, p = 0.04). There were no significant differences in other secondary outcomes. Five participants (2 in the active group, 3 in the sham group) did not complete the 30 day follow-up visit. Among those with evaluable data, approximately 20% had a respiratory-related acute care event at 30 days and were similar in frequency in the two treatment groups.

## Discussion

In this multi-center phase II clinical trial, we found that HFCWO initiated within 24 hours of hospital admission for acute asthma or COPD is associated with high levels of patient adherence and satisfaction. In addition, HFCWO significantly improved dyspnea compared to sham HFCWO, but there were no other significant differences in secondary outcomes between treatment groups.

The high levels of patient adherence and satisfaction in this phase II study establishes the feasibility of HFCWO in this population. Study coordinators assisted patients in the use and activation of pneumatic vests, so it is possible that the high rates of adherence would not occur without such assistance. Without the sham HFCWO control group, we may have erroneously concluded that HFCWO increased patient satisfaction compared to standard medical management alone. Our findings justify the need for sham controls when testing the effect of airway clearance devices on patient-reported outcomes [21].

Nearly twice as many patients treated with active HFCWO reported a clinically significant improvement in dyspnea than with sham HFCWO (71% vs. 42%), which translates into a number needed to treat of approximately 3 (i.e., for every 3 patients treated with active HFCWO, 1 additional patient would report an improvement in dyspnea). These results are unlikely to be explained by reporting bias by the participant or bias in data collection by the research staff, since we employed a sham control group and the study staff who helped participants put on and activate the pneumatic vest were not involved in the collection of outcome data While we did standardize multiple aspects of medical management of acute asthma or COPD, we did not collect data on the use of co-therapies (e.g., use of anti-cholinergic bronchodilators, use of antibiotics), so cannot exclude the possibility that differences in co-therapies contributed to observed differences in dyspnea. However, we believe the likelihood of differences in co-therapies between groups is low, as treating physicians were not permitted to observe the study treatments.

We did not find differences in other secondary outcomes between treatment groups, including those that may be expected to improve with greater airway clearance, such as expectorated sputum volume or airflow obstruction. There are three possible explanations. First, this study may have been underpowered or have had insufficient treatment duration to detect improvements in these other outcomes. Second, we may not have measured markers of airway clearance with adequate precision. Use of spontaneously expectorated sputum volume as an outcome can be problematic due to variability in the ability to expectorate and contamination with saliva. It is also possible that participants may have swallowed sputum or expectorated sputum into containers other than those provided by the research staff. The design of future studies of airway clearance may need to include procedures to assure collection of spontaneously expectorated sputum, to actively encourage cough during and after HFCWO, and to measure wet or dry sputum weight (which may help overcome the effects of dry hospital air on sputum volume). Also, lung volumes and impulse oscillometry may have provided a more sensitive measure of airway clearance[22]. Third, the improvement in dyspnea with HFCWO may have been a type I error.

Nevertheless, results of our study are encouraging and can be used to inform the design of larger-scale, more definitive trials testing the efficacy of HFCWO on clinical endpoints (e.g., feasibility of using HFCWO for acute asthma or COPD, need for a sham-control, need for additional measures of airway clearance). The most common reason for exclusion was the inability to obtain written informed consent from patients. We suspect that patients were concerned about using a pneumatic vest over their chest in a research study during an acute respiratory event. The patient adherence and satisfaction data from this study should be reassuring and may help to facilitate enrollment in future studies. We found that about 1 in 5 patients required acute care for worsening respiratory symptoms within 30 days of hospital discharge; the prevalence of acute care was similar between treatment groups. We employed a limited treatment period (4 treatments spanning 2 calendar days) and found that HFCWO significantly improves dyspnea over this treatment period. Studies using a longer treatment period (e.g., through 30 days post-discharge) are needed to determine if HFCWO improves other clinically meaningful outcomes during the hospitalization (e.g., hospital length of stay), the need for acute care post-discharge, and other outcomes (e.g., local and systemic markers of inflammation, six minute walk distance). Additional, larger studies are also needed to determine which specific patient subgroups (e.g., acute asthma vs. acute COPD; evidence of airway mucus plugging on chest imaging, yes vs. no) are most likely to benefit from HFCWO.

## Conclusions

HFCWO is well tolerated when added to standard medical management in adults hospitalized with acute asthma or COPD and has a large beneficial effect on dyspnea (a number needed to treat of about 3) compared to sham treatment. The high levels of patient satisfaction, including in the sham group, justify the need for sham controls when testing the effect of HFCWO on patient-reported outcomes. Larger studies with a longer treatment period are needed to more fully evaluate the role of HFCWO in improving in-hospital and post-discharge outcomes in this population.

## List of abbreviations

BMI: Body mass index; COAT Trial: Chest Wall Oscillation for Asthma and COPD ExacerbaTions Trial; COPD: Chronic obstructive pulmonary disease; ED: Emergency department; FEV_1 _: Forced expired volume in 1 second; FVC: Forced vital capacity; HFCWO: High frequency chest wall oscillation; Post-BD: Post-bronchodilator.

## Competing interests statement

This was an investigator-initiated study funded by Hill-Rom, Inc. (Principal Investigator: Jerry A. Krishnan, MD, PhD; Co-investigator: Greg Diette, MD, MHS). The sponsor did not participate in the study design, conduct, data analysis, data interpretation, writing of the manuscript, or decisions regarding submission for publication. Other co-authors do not have a potential conflict of interest.

## Authors' contributions

JK and GD conceived of the study and submitted the study proposal for funding to Hill-Rom, Inc. JK and GD contributed substantially to the conduct, data analysis and interpretation, and preparation of this manuscript. JK had full access to the data and will vouch for the integrity of the work as a whole, from inception to published article. AM, UH, VH, SB, EN, AB, AL, VD, SL, and JC each contributed substantially to the conduct, data analysis and interpretation, and preparation of this manuscript. All authors read and approved the final manuscript.
